# Development of Single-Segment Substitution Lines and Fine-Mapping of *qSPP4* for Spikelets Per Panicle and *qGW9* for Grain Width Based on Rice Dual-Segment Substitution Line Z783

**DOI:** 10.3390/ijms242417305

**Published:** 2023-12-09

**Authors:** Keli Deng, Han Zhang, Jiayi Wu, Zhuowen Zhao, Dachuang Wang, Guangyi Xu, Jinjin Yu, Yinghua Ling, Fangming Zhao

**Affiliations:** Rice Research Institute, Academy of Agricultural Science, Engineering Research Center of South Upland Agriculture, Ministry of Education, Southwest University, Chongqing 400715, China; 15520114914@163.com (K.D.); 18809864123@163.com (H.Z.); 13880468288@163.com (J.W.); 13083810503@163.com (Z.Z.); wangdachuan6@163.com (D.W.); 15520096684@163.com (G.X.); 15320218356@163.com (J.Y.); lingyh65@swu.edu.cn (Y.L.)

**Keywords:** chromosomal segment substitution line, grain number and size, QTL, additive effect

## Abstract

Single segment substitution line (SSSL) libraries are an ideal platform for breeding by design. To develop SSSLs-Xihui18 covering the whole genome, a novel rice chromosome segment substitution line (CSSL), Z783, carrying two substitution segments (average length of 6.55 Mb) on Chr.4 and Chr.9 was identified, which was a gap in the library previously. Z783 was developed from the progeny of recipient “Xihui18” (an *indica* restorer line) and donor “Huhan3” (a *japonica* cultivar) by advanced backcross combined molecular marker-assisted selection (MAS). It displayed multiple panicles and less spikelets and wide grains. Then, a F_2_ population derived from Xihui18/Z783 was used to map quantitative trait loci (QTLs) for yield-related traits by the mixed linear model method. Nine QTLs were detected (*p* < 0.05). Furthermore, three SSSLs were constructed by MAS, and all 9 QTLs could be validated, and 15 novel QTLs could be detected by these SSSLs by a one-way ANOVA analysis. The genetic analysis showed that *qSSP4* for less spikelets and *qGW9* for wide grain all displayed dominant gene action in their SSSLs. Finally, *qSSP4* and *qGW9* were fine-mapped to intervals of 2.75 Mb and 1.84 Mb, on Chromosomes 4 and 9, respectively. The results lay a solid foundation for their map cloning and molecular breeding by design.

## 1. Introduction

Global food production needs to double by 2050 to meet the demand of the swiftly growing population [1,2]. Rice is a staple food for more than half of the world’s populations [3], and its yield is of great importance to human security and development [4]. Rice yield is affected by three components: grain weight, grain number per panicle, and panicle number per plant. The grain number per panicle is determined by the number of primary and secondary branches, and the grain weight is determined by the grain size and also the rate of grain filling [5]. The grain size is then measured by the grain length, width, and thickness, which are controlled by multiple genes [6].

Chromosome segment substitution lines (CSSLs) are valuable pre-breeding tools for broadening the genetic base of existing cultivars and harnessing the genetic diversity from wild and distantly related species, and powerful tools for enhancing the underlying basis of genetic analysis and identifying naturally occurring favorable alleles in unadapted germplasms. Each line in a CSSL library has one or several specific marker-defined large donor segments [7,8]. A CSSL, when carrying only one single substitution segment, is called a single segment substitution line (SSSL) [9], or near-isogenic line (NIL) [10,11]. Until now, CSSLs derived from the distant relatives of rice including *O*. *meridionalis*, *O. glumepatula*, *O. rufipogon,* and *O. glaberrima* have been constructed by different institutions [8,12,13]. The Mendelization of QTLs is best accomplished by the construction of SSSLs or NILs ideally differing only for the alleles in a small genomic region spanning a few centi-Morgans (cM) around the QTL of interest. SSSLs or NILs allow further fine mapping and chromosome walking to start the physical mapping of a target locus and finally accomplish the positional cloning of genes at the QTL [9,10,11,14,15].

In recent years, several QTLs for grain size have been identified by NILs or SSSLs, the function of which involves various signaling pathways, including the ubiquitin–proteasome pathway, G-protein signaling, the mitogen-activated protein kinase signaling pathway, phytohormones and transcriptional regulatory factors [16]. Ubiquitination is an enzymatic post-translational modification, and ubiquitin proteins contain 76 amino acids bound to target proteins via covalent bonds, and they are acted upon sequentially by three unique enzymes (E1, E2, and E3) [17]. *GW2* encodes a RING-type E3 ubiquitin ligase with ubiquitination and auto-ubiquitination activity located in the cytoplasm and nucleus [18,19]. *GW2* negatively regulates the rice grain size in the nucleus by causing the ubiquitination-mediated degradation of expansin-like 1(EXPLA1) [20,21]. The heterotrimeric G-protein complex is a conserved molecular signaling mechanism that transmits signals from transmembrane receptors to downstream targets and contains three subunits, Gα, Gβ, and Gγ [22]. The QTL for grain weight (*GW6a*) encodes a new-type GNAT-like protein with intrinsic histone acetyltransferase activity (*OsglHAT1*) [23,24]. The MAPK pathway is activated by different intra and extracellular signals that regulate various life activities. The GSN1-MAPK module integrates cellular nutrient growth and reproductive growth to regulate the relationship between the grain number and grain size [25]. Plant hormones play multiple roles in regulating the various stages of plant growth and development. Brassinosteroids (BRs) are an important phytohormone in rice growth regulation and regulate the development of many agronomic traits. *GLW10*/*OsBSK2* directly interacts with *OsBRI1* and may regulate the seed size alone without going through the BR signaling pathway [26]. *TGW6* for thousand-grain weight encodes IAA-glucose hydrolase, which regulates the transition from the syncytial to the cellular phase during early endosperm development by regulating IAA supply [27]. Transcription factors have been found to take part in regulating the grain size. *GW8* for grain width encodes a transcription factor with an SBP domain, *OsSPL16*, and it increases the grain width and yield by promoting cell division and grain filling [28].

The grain number per panicle formation is a complex biological process that is regulated by multiple genes, involving the development of axillary meristematic tissues, inflorescence structural completion, and seed development [29]. Several plant hormones are closely related to panicle development and grain number formation, such as auxin, cytokinin, gibberellin, jasmonic acid, ethylene, and brassinosteroids [30]. *GN1a* for grain number encodes *OsCKX2* and its reduced expression leads to the accumulation of cytokinins in the inflorescence meristems, thereby increasing the number of grains in the spike and improving the yield [31]. The QTL *GNP1* encodes GA20ox1, which increases the grain number and yield by increasing the cytokinin activity in rice panicle meristems [32]. In addition, there are genes in other pathways that regulate the grain number development. *OsER1* (*ERECTA 1*) encodes a receptor-like protein kinase that is a negative regulator of the grain number per panicle. It acts upstream of the *OsMKKK10-OsMKK4-OsMPK6* cascade signaling pathway and regulates the phosphorylation level of *OsMPK6*, which in turn participates in the morphogenesis of the rice spike by the regulation of cytokinin metabolism [25].

Although we understand some genetic mechanisms of the regulation of the grain size and grain number by cloning related QTLs, there are a great deal of micro-effect genes for these traits which have not been identified, and also the knowledge gap, rationale, and hypothesis need more interpretation. Thus, it is very necessary to detect novel genes for grain number and grain size. Particularly, novel genes identified by SSSL are more favorable to achieve breeding by design in the whole genome of rice. Here, we prepared to identify novel genes for these traits on Chromosomes 4 and 9 and develop their SSSLs by a dual-segment substitution line, Z783, in the genetic background of Xihui18. The findings will be important to explore novel genes for grain size and grain number and then to broaden the library of Xihui18-SSSLs for breeding by design.

## 2. Results

### 2.1. Identification of Substitution Segments in Z783

**Figure 1:**
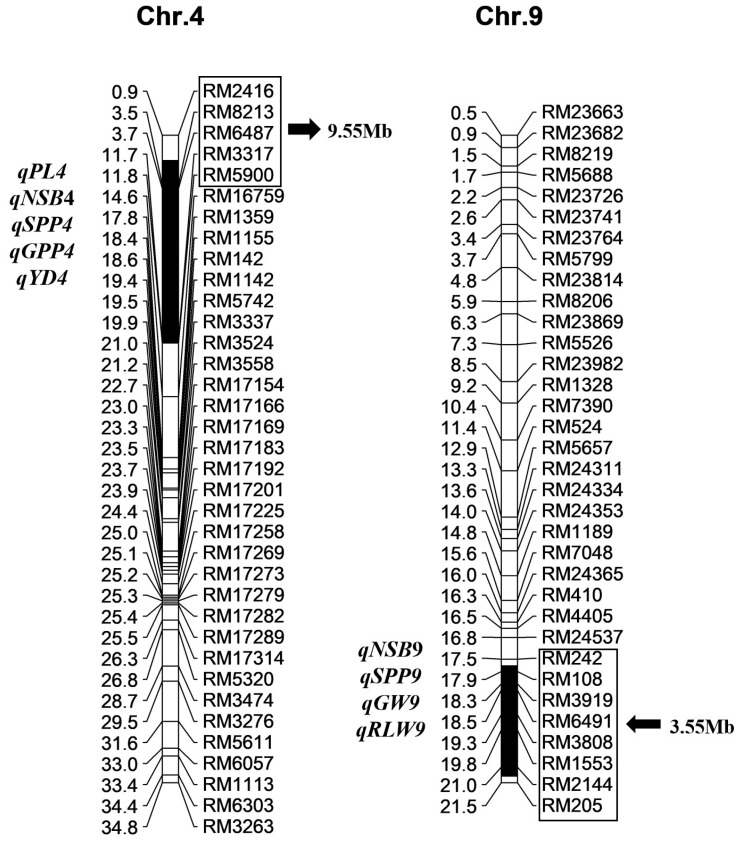
Substitution segments and detected QTLs in Z783 (*Indica* 9311 as the referred genome). Physical distances (Mb) and mapped QTLs are marked at the left of each chromosome; markers and substitution length (black arrow direction) are displayed to the right of each chromosome. PL, panicle length; NSB, number of secondary branches; GPP, grain number per panicle; GW, grain width; RLW, ratio of length to width; YD, yield per plant.

Based on the previous development of CSSL Z783 with two substitution segments, nine SSR markers in the substitution segments and 36 outside them from 12 chromosomes were used to detect the target segments and the purity of the genetic background of Z783 using 10 plants. The results showed that the substitution segments from 10 plants in Z783 were consistent, no other residual segments from donor Xihui18 were detected, and the substitution segments of Z783 were accurately identified. The two substitution segments in Z783 from Huhan3 were distributed on rice Chromosomes 4 and 9, and the estimated lengths of the substitution segments were 9.55 Mb and 3.55 Mb, respectively, with an average substitution length of 6.55 Mb (Figure 1).

### 2.2. Phenotype Analysis of Z783

**Figure 2:**
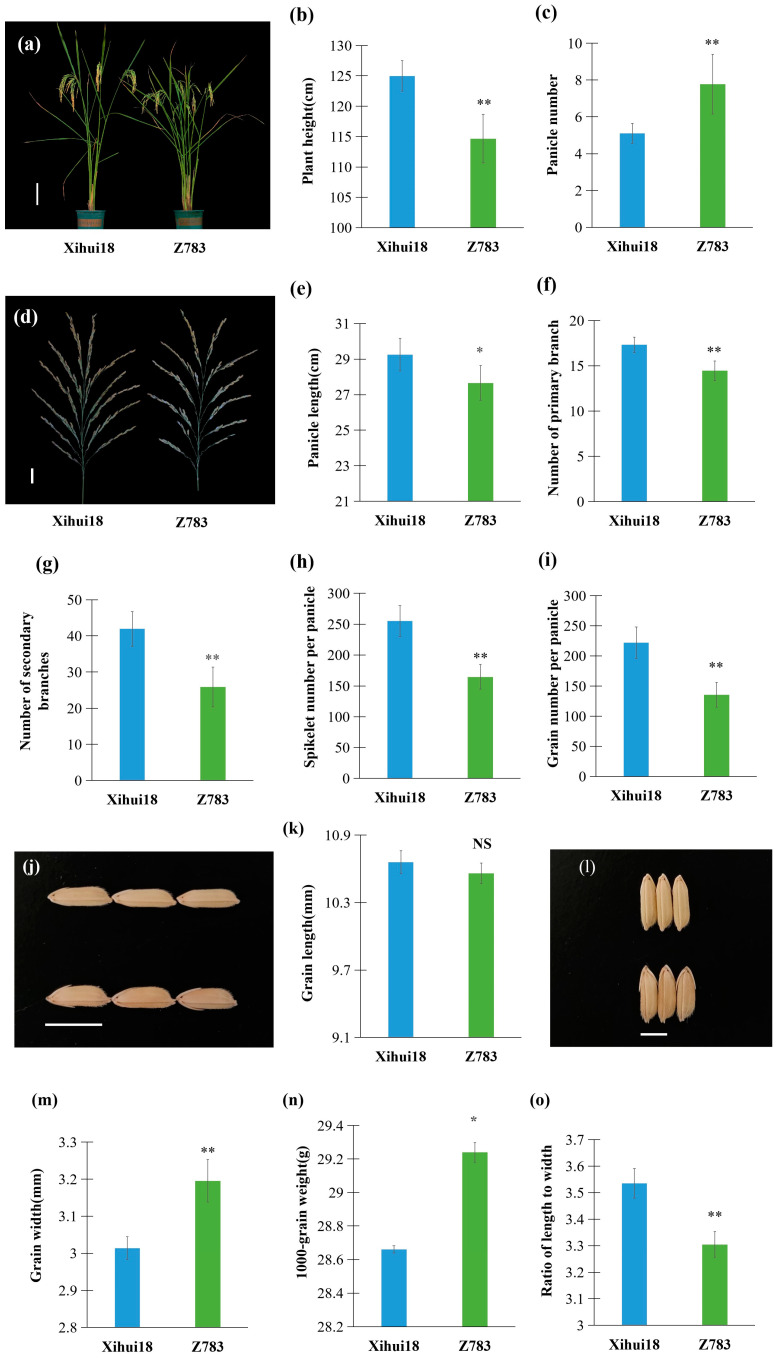
Phenotype analysis of Xihui18 and Z783. (**a**) Plant types of Xihui18 and Z783; (**b**,**c**) statistic analysis of plant height (**b**) and panicle numbers (**c**) between Xihui18 and Z783; (**d**) panicles of Xihui18 and Z783; (**e**–**i**) statistic analysis of panicle length (**e**), number of primary branches (**f**), number of secondary branches (**g**), spikelet number per panicle (**h**), grain number per panicle (**i**) between Xihiui18 and Z783; (**j**) grain length of Xihui18 (up) and Z783 (down); (**k**) statistic analysis between grain length of Xihui18 and Z783; (**l**) grain width of Xihui18 (up) and Z783 (down); (**m**–**o**) statistic analysis of grain width (**m**), 1000 grain weight (**n**), and ratio of length to width (**o**) between Xihui18 and Z783. Bars represented 20 cm in (**a**), 5 cm in (**d**), 10 mm in (**j**), and 5 mm in (**l**). * and ** indicate significant differences at the 0.05 and 0.01 level, respectively by *Student-t* test.

Compared with the recipient Xihui18, Z783 was a similar plant type (Figure 2a), belonging to near-isogenic lines. However, the plant height of Z783 was significantly shorter than that (124.99 cm) of Xihui18, by 8.3% (Figure 2a,b). The panicle number per plant of Z783 was significantly more than that (5.1) of Xihui18, by 53% (Figure 2a,c). The panicle length, number of primary branches, number of secondary branches, spikelet number per panicle, and grain number per panicle displayed less than those of Xihui18, by 5.4%, 16.6%, 38.2%, 35.4%, and 38.8%, respectively (Figure 2d–i). The grain length (10.56 mm) of Z783 exhibited no significant difference from that (10.66 mm) of Xihui18 (Figure 2j,k). The grain width (3.20 mm) and 1000 grain weight (29.24 g) of Z783 were greater than those of Xihui18, by 6.02% and 2.00%, respectively (Figure 2l–n), while the ratio of the length to width (3.3) of Z783 was significantly lower than that of Xihui18, by 6.5% (Figure 2o), and there were no significant differences observed in the seed setting rate, and the yield per plant between Z783 and Xihui18.

### 2.3. Identification of QTL by a Secondary F_2_ Population from Xihui18/Z783

In the secondary F_2_ segregating population derived from the cross between Xihui18 and Z783, a total of nine QTLs for yield-related traits were identified, distributed on the fourth and ninth chromosomes, explaining 8.05% to 17.96% of the phenotypic variation (Table 1). These QTLs included one for the panicle length, two for the number of secondary branches, two for the spikelet number per panicle, one for the grain number per panicle, one for the grain width, one for the ratio of the length to width, and one for the yield per plant. Among them, the additive effects of seven QTLs (*qPL4*, *qNSB4*, *qNSB9*, *qGPP4*, *qGW9*, *qRLW9,* and *qYD4*) explained more than 10% of the variation. *qPL4* for the panicle length, *qNSB4* for the number of secondary branches, *qSPP4* for the spikelet number per panicle, *qGPP4* for the grain number per panicle, and *qYD4* for the yield per plant were all linked to the same marker, RM3317, of Chromosome 4, the additive effects of which from Huhan3 decreased the corresponding traits by 0.51 cm, 2.84, 10.75, 11.92, and 3.93 g, respectively, explaining 12.13%, 13.07%, 8.05%, 10.51%, 12.96%, of the phenotypic variation, respectively. *qNSB9*, *qSPP9*, *qGW9,* and *qRLW9* were all linked to the same marker, RM2144, of Chromosome 9, and their additive effects from Huhan3 decreased the number of secondary branches by 2.51, the spikelets number per panicle by 11.78, and the ratio of the length to width by 0.04 for *qNSB9*, *qSPP9,* and *qRLW9*, while the additive effect of *qGW9* from Huhan3 increased the grain width by 0.04 mm. This explained the phenotypic variations by 10.27%, 9.67%, 17.96%, and 11.60%, respectively.

### 2.4. Construction of SSSLs and Additive Effects Analysis of QTLs Identified by the SSSLs

**Figure 3:**
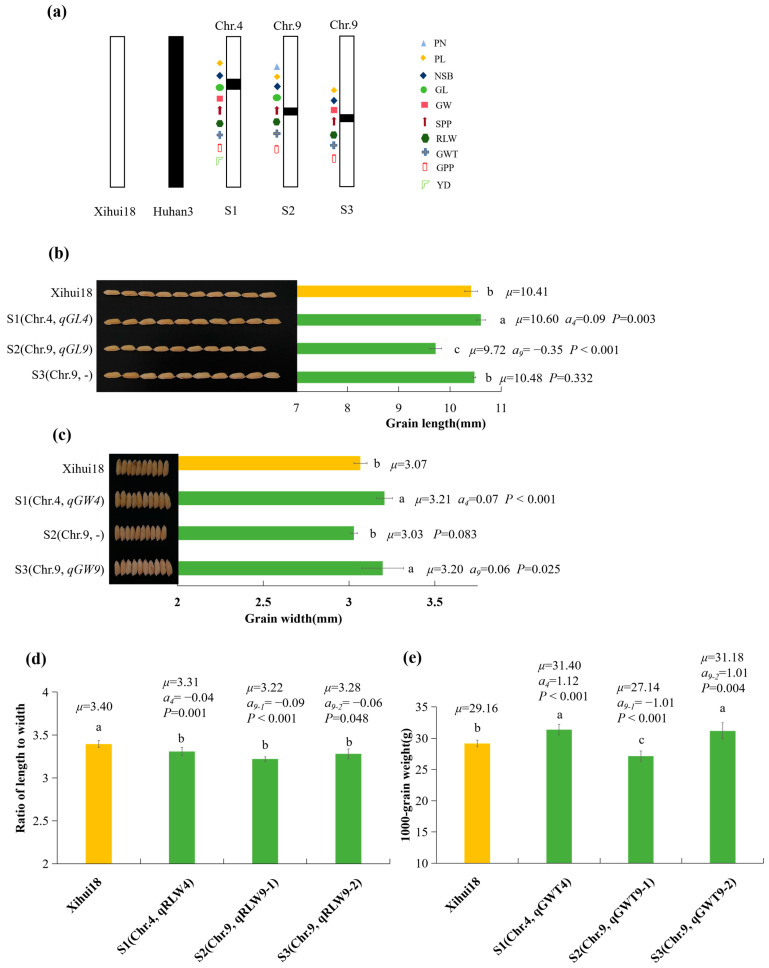
Substitution segment of the development of SSSLs (S1–S3) and additive effects analysis of QTL for grain size. (**a**) diagram of the locations of substitution segments and QTL in S1–S3. (**b**–**e**) Parameters of QTL for grain size in different SSSLs. (**b**) Grain length; (**c**) grain width; (**d**) ratio of length to width; (**e**) 1000 grain weight. Different lowercase letters on each top column indicated significant difference (*p* < 0.05), as determined by Duncan’s multiple comparison. *μ*, the average value of each line; *a_i_*, additive effect for each QTL controlling the trait, the positive value of which showed allele from substitution segment increasing phenotypic value, while negative value decreased. *p* < 0.05 in SSSL indicated that a QTL existed in the substitution segment of the SSSL, as determined by one-way ANOVA and LSD multiple comparison with Xihui18. S1 (Chr.4: RM2416 (0.90 Mb)--RM3317 (11.70 Mb)--RM5900 (11.84 Mb)); S2 (Chr.9: RM242 (17.48 Mb)--RM108 (17.93 Mb)--RM2144 (21.02 Mb)); S3 (Chr.9: RM108 (17.93 Mb)--RM2144 (21.02 Mb)--RM205 (21.53 Mb)). The middle markers indicated the substitution segment from the donor, whereas the markers at each end of the substitution segment linked with “--” indicated that segment recombination might have occurred.

Based on the QTL mapping results, three SSSLs (S1–S3) were further developed in the F_3_ population by MAS. S1 harbored the substitution segment RM2416--RM3317--RM5900 of Chromosome 4. S2 carried the substitution segment RM242--RM108--RM2144 of Chromosome 9. S3 contained the substitution segment RM108--RM2144--RM205 of Chromosome 9 (Figure 3a).

Furthermore, the QTLs for the yield-related traits was detected by these SSSLs (S1–S3). The result showed that all nine QTLs (*qPL4*, *qNSB4*, *qNSB9*, *qSPP4*, *qSPP9*, *qGPP4*, *qGW9*, *qRLW9,* and *qYD4*) mapped in the secondary F_2_ population could be validated by S1-S3. Among them, *qNSB9*, *qSPP9*, and *qRLW9* mapped in the F_2_ population were consistent with *qNSB9-2*, *qSPP9-2*, and *qRLW9-2* detected in S3, respectively. In addition, 15 novel QTLs (*qPN9*, *qPL9-1*, *qPL9-2*, *qNSB9-1*, *qSPP9-1*, *qGPP9-1*, *qGPP9-2*, *qGL4*, *qGL9*, *qGW4*, *qRLW4*, *qRLW9-1*, *qGWT4*, *qGWT9-1,* and *qGWT9-2*) were detected by S1, S2, and S3. These results revealed that single segment substitution lines had a higher efficiency in QTL detection (Figure 3a) than in the F_2_ population from Xihui18/Z783 (Table 1). The grain length (10.60 mm) of S1 carrying *qGL4* (a = 0.09 mm) was significantly longer than those (10.41 and 10.48 mm) of Xihui18 and S3 without the QTL for GL. While the grain length (9.72 mm) of S2 harboring *qGL9* (a = −0.35 mm) was significantly shorter than that (10.41 and 10.48 mm) of Xihui18 and S3 without the grain length QTL (Figure 3b). The grain width (3.21 and 3.20 mm) of S1 harboring *qGW4* (a = 0.07 mm) and S3 containing *qGW9* (a = 0.06 mm) were significantly broader than that of Xihui18 (3.07 mm) and S2 without the QTL for GW (Figure 3c). The ratios of the length to width (3.31, 3.22 and 3.28) of S1 with *qRLW4* (a = −0.04), S2 containing *qRLW9-1* (a = −0.09), and S3 harboring *qRLW9-2* (a = −0.06) were significantly less than that (3.40) of the recipient parent Xihui18 (Figure 3d). The 1000 grain weight (31.40 g, 31.18 g) of S1 containing *qGWT4* (a = 1.12 g) and S3 harboring *qGWT9-2* (a = 1.01 g) were significantly heavier than that (29.16 g) of Xihui18, while the 1000 grain weight (27.14 g) of S2 carrying *qGWT9-1* (a = −1.01 g) were lighter significantly than that (29.16 g) of Xihui18 (Figure 3e).

**Figure 4:**
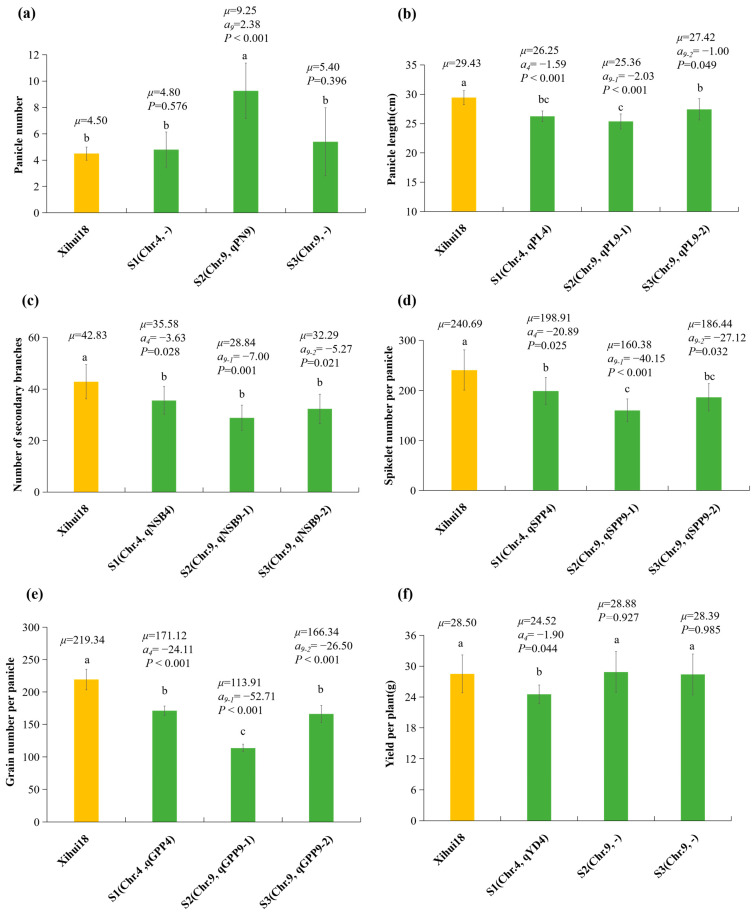
Additive effects analysis of QTL for panicle-related traits. (**a**) Panicle number per plant; (**b**) panicle length; (**c**) number of secondary branches per panicle; (**d**) spikelet number per panicle; (**e**) grain number per panicle; (**f**) yield per plant. Different lowercase letters on each top column indicated significant difference (*p* < 0.05), as determined by Duncan’s multiple comparison. *μ*, the average value of each line; *a_i_*, additive effect for each QTL controlling the trait, the positive value of which showed allele from substitution segment increasing phenotypic value, while negative value decreased one. *p* < 0.05 in SSSL indicated that a QTL existed in the substitution segment of the SSSL, as determined by one-way ANOVA and LSD multiple comparison with Xihui18. S1 (Chr4: RM2416 (0.90 Mb)--RM3317 (11.70 Mb)--RM5900 (11.84 Mb)); S2 (Chr9: RM242 (17.48 Mb)--RM108 (17.93 Mb)--RM2144 (21.02 Mb)); S3 (Chr9: RM108 (17.93 Mb)--RM2144 (21.02 Mb)--RM205 (21.53 Mb)). The middle markers indicated the substitution segment from the donor, whereas the markers at each end of the substitution segment linked with “--” indicated that segment recombination might have occurred.

S2 containing *qPN9* (a = 2.38) displayed a significant increase in the panicle number (9.25 per plant) compared to that (4.50) of Xihui18. However, S1 and S3 without the QTL for *PN* did not exhibit a significant difference in the panicle numbers (4.8 and 5.4 per plant) compared to Xihui18 (4.13) (Figure 4a). The panicle length (26.25, 25.36, and 27.42 cm) of S1 containing *qPL4* (a = −1.59 cm), S2 harboring *qPL9-1* (a = −2.03 cm), and S3 carrying *qPL9-2* (a = −1.00 cm) were significantly less than that (29.43 cm) of Xihui18 (Figure 4b). The number of secondary branches per panicle (35.58, 28.84, 32.29) of S1 harboring *qNSB4* (a = −3.63), S2 containing *qNSB9-1* (a = −7.00), and S3 carrying *qNSB9-2* (a = −5.27) were significantly lower than that (42.83) of Xihui18 (Figure 4c). The spikelet numbers per panicle (198.91, 160.38, 186.44) were significantly decreased in S1 carrying *qSPP4* (a = −20.89), S2 containing *qSPP9-1* (a = −40.15), and S3 carrying *qSPP9-2* (a = −27.12) compared to that (240.69) of Xihui18 (Figure 4d). The grain numbers per panicle (171.12, 113.91, and 166.34) of S1 with *qGPP4* (a = −24.11), S2 carrying *qGPP9-1* (a = −52.71), and S3 containing *qGPP9-2* (a = −26.50) were less than that (219.34) of Xihui18 (Figure 4e). The yield per plant (24.52 g) of S1 carrying *qYD4* (a = −1.92 g) was significantly less than that (28.50 g) of Xihui18, whereas the yield per plant (28.88 g and 28.39 g) of S2 and S3 without for the trait displayed no significant difference to that (28.50 g) of Xihui18 (Figure 4f).

### 2.5. Genetic Analysis of qGW9 for Grain Width and qSPP4 for Spikelet Number per Panicle

**Figure 5:**
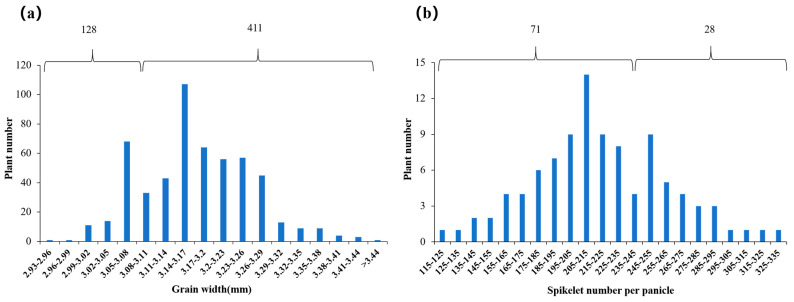
Genetic analysis of *qGW9* for grain width and *qSPP4* for spikelet number per panicle. (**a**) Grain width distribution in the F_3_ population consists of 539 individual plants derived from a recombinant plant with *qGW9*. (**b**) Spikelet numbers of main panicle distribution in the F_3_ population consists of 99 individual plants derived from a recombinant plant with *qSPP4*.

The donor parent Huhan3 exhibits a wide grain type and lower number of spikelets per panicle, while the recipient parent Xihui18 exhibits a narrow grain type and multiple numbers of spikelets per panicle. The Z783 carrying two substitution segments from Huhan3 in its genetic background displayed less spikelets and wide grains. Based on the QTL mapping of the F_2_ population from Xihui18/Z783, the two F_3_ populations were further developed by two recombinant plants of a single *qSPP4* locus and *qGW9* locus, respectively. In the F_3_ population consisting of 539 individuals with *qGW9*, the grain width exhibited a bimodal distribution, one peak for a narrow grain from 2.93 mm to 3.11 mm, with 128 plants, and the other one for wide grain from 3.11 to 3.44 mm, with 411 individuals (Figure 5a). Then, the chi-square test showed that the numbers of wide-grain (411) and narrow-grain individuals (128) corresponded to a separation ratio of 3:1 (*χ*^2^ = 0.39 < *χ*^2^ _(0_._05,1)_ = 3.84). The results indicated that *qGW9* controlling a wide grain from Huhan3 exhibited a single dominant gene action in the single segment substitution line S3 (Figure 5a). 

In the F_3_ population consisting of 99 individuals with *qSPP4*, the number of spikelets of the main panicle exhibited a bimodal distribution, one peak for lower spikelet numbers from 115 to 245, with 71 plants, and the other one for multiple spikelet numbers from 245 to 335, with 28 plants (Figure 5b). According to the chi-square test analysis, the ratio of plant numbers with fewer spikelets (71) to the plant number with multiple spikelets (28) fitted a 3:1 segregation (*χ*^2^ = 0.52< *χ*^2^_(0.05, 1)_ = 3.84). The result showed that *qSPP4* controlling the feature of less spikelets, from Huhan3, also displayed single dominant gene inheritance in the single segment substitution line S1 (Figure 5b). 

### 2.6. Fine Mapping of qGW9 for Wide Grain 

**Figure 6:**
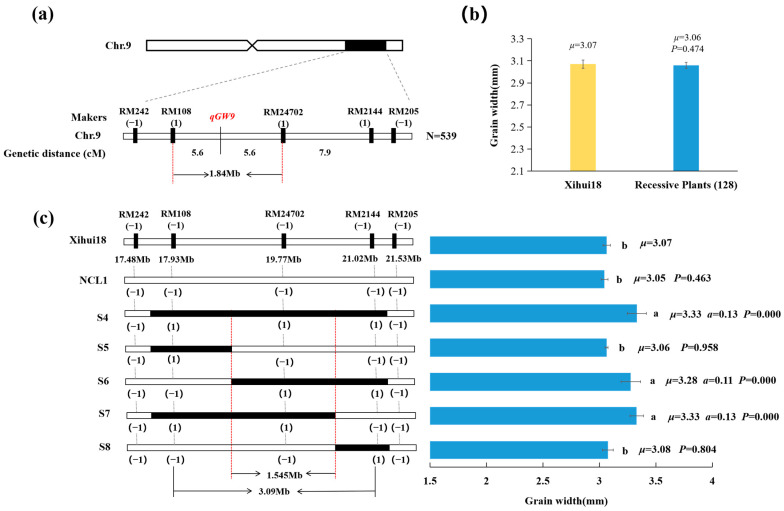
Fine mapping and substitution mapping of *qGW9.* (**a**) *qGW9* has been fine-mapped to the interval between RM108 and RM24792. (**b**) Statistic analysis of grain widths for 10 individuals of Xihui18 and 128 recessive individuals. (**c**) Black regions indicate the estimated length of the substitution segment. NCL, negative control line, the bands of markers in which in corresponding substitution intervals was consistent with those of Xihui18. Different lowercase letters on each top column indicated significant difference (*p* < 0.05), as determined by Duncan’s multiple comparison.

Due to *qGW9* for wide grain from Huhan3 being a single dominant gene in S3, we further fine-mapped *qGW9* using 128 recessive plants in the F_3_ population developed by a recombinant plant with a single *qGW9* locus (Figure 6a). The grain width (3.06 mm) of 128 recessive showed no significant difference from that (3.07 mm) of Xihui18 (Figure 6b). This enabled *qGW9* to be fine-mapped between RM108 and RM24702 on Chromosome 9, with a physical distance of 1.84 Mb (Figure 6a). Moreover, we constructed a series of secondary single segment substitution lines S4–S8 for substitution mapping of *qGW9* (Figure 6c). The grain width (3.05 mm) of the negative control line (NCL1), the bands of markers of which in corresponding substitution intervals was consistent with those of Xihui18, displaying no significant difference with that (3.07 mm) of Xihui18. Besides, S5 harboring the RM108 substitution segment and S8 carrying the RM2144 substitution segment exhibited no differences in the grain width from that of Xihui18, while S4 harboring RM108-RM24702-RM2144, S6 carrying RM24702-RM2144, and S7 containing RM108-RM24702 displayed a significantly wider grain than Xihui18. Thus, according to the theory of substitution mapping, *qGW9* should be in 1.545 Mb of the estimated length of RM108-RM24702-RM2144 (Figure 6c). This result is consistent with the fine mapping of *qGW9* (Figure 6a). These results showed that *qGW9* was near RM24702, with an estimated length of 1.545 Mb, between the markers RM108 to RM24702.

### 2.7. Fine Mapping of qSPP4 for Spikelets per Panicle 

**Figure 7:**
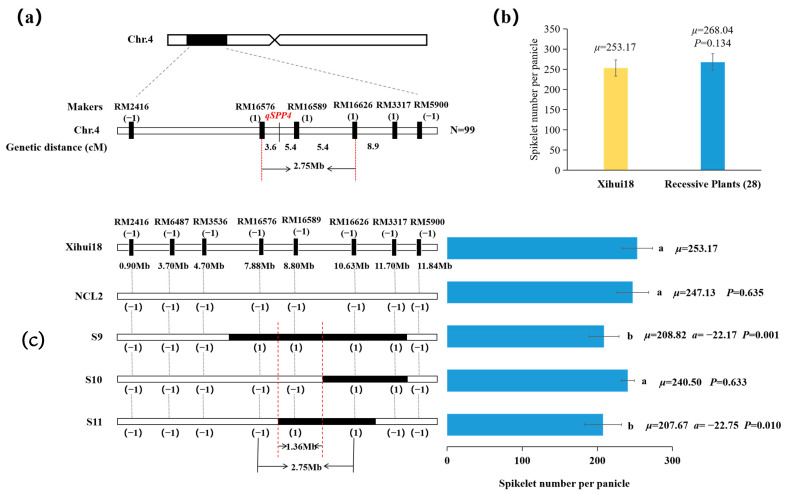
Fine mapping and overlapping substitution mapping of *qSPP4.* (**a**) *qSPP4* has been fine-mapped to the interval between RM2416 and RM5900. (**b**) Statistic analysis of spikelet number per panicle for 10 individuals of Xihui18 and 28 recessive individuals with multiple spikelets. (**c**) Black regions indicate the estimated length of the substitution segment. NCL, negative control line, the bands of markers of which in corresponding substitution intervals was consistent with those of Xihui18. Different lowercase letters on each top column indicated significant difference (*p* < 0.05), as determined by Duncan’s multiple comparison.

Due to *qSPP4* for less spikelets per plants from Huhan3 being a single dominant gene in S1, we further fine-mapped *qSPP4* using 28 recessive plants in the F_3_ population developed by a recombinant plant with a single *qSPP4* locus (Figure 7a). The number of spikelets of the main panicle (268.04) of 28 recessive individuals showed no significant difference from that (253.17) of Xihui18 (Figure 7b). This enabled *qSPP4* to be fine-mapped between RM16576 to RM16626 on Chromosome 4, with a physical distance of 2.75 Mb (Figure 7a). Moreover, we constructed three secondary single segment substitution lines, S9-S11, for the substitution mapping of *qSPP4* (Figure 7c). The spikelets number (247.13) of the main panicle in NCL2 was not significantly different from that (253.17) of Xihui18. Furthermore, the spikelet number (240.50) of the main panicle in S10 harboring RM16626-RM3317 was not significantly different from that (253.17) in Xihui18, indicating the substitution interval did not carry *qSPP4*. Whereas the spikelet numbers (208.82 and 207.67) of the main panicle in S9 carrying RM16576-RM16589-RM16626-RM3317 and S11 harboring RM16589-RM16626 were both significantly less than that (253.17) of Xihui18. Thus, according to the theory of substitution mapping, *qSPP4* should be in the estimated length of 1.36 Mb between RM16576 and RM16626 and 2.75 Mb of the largest estimated length (Figure 7c), which was consistent with the result (2.75 Mb) of the fine-mapping of *qSPP4* (Figure 7a).

## 3. Discussion

### 3.1. The 11 Single Segment Substitution Lines Dissected from CSSL-Z783 Has Important Application Value in Rice Breeding by Design

The utilization of intra-subspecific heterosis has reached its limit owing to their close relationship each other. The use of heterosis between subspecies and wild species will be imperative due to their strong heterosis from their distant genetic relationship. However, direct subspecies crosses often cause sterility and a reducing seed setting rate [33]. CSSLs can overcome this problem. They are not only a powerful genetic resource for surveying the genetic potential of donor germplasms, but are also valuable pre-breeding tools for broadening the genetic base of existing cultivars from the wild and distantly related species [7,34]. In this study, we constructed an inter-subspecies-type rice CSSL, Z783, which contained two substitution segments from *japonica*-type Huhan3 in the genetic background of *indica*–type Xihui18. Xihui18 was an elite rice *indica* restorer line with a strong combining ability, the CMS type of which being used as a restorer comprised the restoring genes *Rf1*, *Rf3* (Chr.1), *Rf2* (Chr.2), and *Rf4* (Chr.10) [35,36,37]. Xihui18 exhibited a long panicle with multiple grains, and long, narrow grain. However, its number of panicles per plant was fewer (4.8 for average in 2021 and 2023). Compared with Xihui18, Z783 displayed a significantly wider grain and a significantly increasing panicle number per plant. However, its grain-related traits were significantly decreased. Thus, it was still not an ideal breeding germplasm due to carrying two substitution segments with unknown QTLs. In order to create more ideal materials for direct breeding and basic study in gene functional analysis, 11 single segment substitution lines were further developed carrying QTLs for various yield-related traits. S2 harboring *qGL9* (a = −0.35 mm), *qGWT9-1* (a = −1.01 g), and *qPN9* (a = 2.58) displayed a shorter, lighter grain (27.14 g) and multiple panicles (9.25) per panicle than that (29.16 g and 4.50) of Xihui18. However, the yield per plant of S2 exhibited no difference to Xihui18 due to carrying negative QTLs for *qGWT4* and *qGPP9-2* (a = −52.71) for the grain number per plant. S3 carrying *qGW9* (a = 0.06 mm) and *qGWT9-2* (a = 1.01 g) displayed a wider, larger grain (31.08 g) than that (29.16 g) of Xihui18. Also, the yield per plant (28.39 g) of S3 showed no difference from that (28.50 g) of Xihui18 due to carrying a negative QTL for *qGPP9-2* (a = −26.50). S1 displayed a longer and heavier grain (10.60 mm for GL, 3.20 mm for grain width and 31.4 g for 1000-GWT) than that of Xihi18 due to carrying the positive effects of *qGL4*, *qGW4,* and *qGWT4*. Whereas, S1 also harbored the negative effects of QTLs for GPP and YD. The other eight SSSLs were developed in the overlapping substitution mapping of *qGW9* and *qSPP4*, the information of which was not incomplete. These SSSLs will be a potentially valuable genetic resource for rice breeding. The imperfection of these SSSLs in terms of the lower grain number could be remedied by pyramiding QTLs for increasing the grain number of SSSL-Xihui18 or combined with sterile lines with multiple grains per panicle. Thus, a new cultivar should be produced. Furthermore, 27 SSSLs-Xihui18 have been developed, the substitution segments of which from donor Huhan3 and Jinhui35 are distributed across 11 chromosomes except the Chr.10 [38,39,40,41,42]. Our dissected SSSLs together with these previously developed SSSLs will be important for breeding by design in rice yield improvement. Some SSSLs have been used to design a series of novel cultivars successfully, such as Huaxiaohei1 (Guangdong Rice 2005015) and Huabiao1 (Guangdong Rice 2009033) [43]. These achievements suggest that using SSSLs as a platform can facilitate designing and breeding cultivars to meet production needs.

### 3.2. Comparison of QTLs Identified in the Study with the Reported Genes

In total, 24 QTLs for the yield-related traits were identified in both the secondary F_2_ population from Xihui18/Z783 and the isolated three secondary single-segment substitution lines. By comparing with the previously cloned genes, *OsmiR530* was found to be in the substitution intervals of *qPL4*, *qNSB4*, *qGPP4*, *qSPP4*, *qGL4*, *qGW4*, *qYD4,* and *qGWT4*. They are all linked to the same marker, RM3317. *OsPIL15* activates *OsmiR530* expression by directly binding to the G-box elements in the promoter, and then targets *OsPL3* to negatively regulate the rice yield. *OsmiR530* overexpression significantly decreases the grain size and panicle branching, leading to yield loss [44]. Thus, *OsmiR530* can act as the candidate gene for *qPL4*, *qNSB4*, *qGPP4*, *qSPP4* (related to panicle branching), and *qGL4*, *qGW4*, *qRLW4*, *qGWT4* (related to grain size), and *qYD4*, which might belong to pleiotropy. Next, *qNSB9-2*, *qSPP9-2*, *qPL9-2,* and *qGPP9-2* were all linked to the same marker, RM2144. *OsSPL18* and *OsSHI1* were located in the substitution intervals. *OsSPL18* is cleaved by *OsmiR156k*, and then regulates panicle development by positively regulating the expression of *DEP1.* The results uncovered a new *OsmiR156k*-*OsSPL18*-*DEP1* pathway regulating the grain number in rice [45]. *OsSHI1* represses the transcriptional activation activity of *IPA1* by affecting its DNA binding activity towards the promoters of both *OsTB1* and *OsDEP1*, resulting in an increased tiller number and diminished panicle size [46]. Therefore, *OsSPL18* and *OsSHI1* can be considered as candidate genes for *qNSB9-2*, *qSPP9-2*, *qPL9-2,* and *qGPP9-2*; however, *qGW9*, *qRLW9-2,* and *qGWT9-2* related to grain size have been previously unreported, although they are located in the same interval of the above QTLs. Furthermore, *qGL9, qRLW9-1,* and *qGWT9-1* were all linked to the same marker, RM108, from which *OsISA3* is 0.07 Mb away. The simultaneous overexpression of *OsISA3* and *OsBMY4*, the key β-amylase and debranching enzymes, notably increased the grain length by accelerating the starch degradation efficiency and increasing the sugar supply for sink organs, which is probably the direct cause of the increase in the 1000 grain weight [46]. Thus, *OsISA3* should be a candidate gene for *qGL9*, *qRLW9-1,* and *qGWT9-1*. Although *qPN9*, *qPL9-1*, *qNSB9-1*, *qSPP9-1,* and *qGPP9-1* are also located in the same interval as the above QTLs related to the grain size, these QTLs related to the panicle number and grain number have not been reported.

In summary, eight QTLs (*qGW9*, *qRLW9-2*, *qGWT9-2*, *qPN9*, *qPL9-1*, *qNSB9-1*, *qSPP9-1,* and *qGPP9-1*) have not yet been cloned, and may be previously unreported QTLs. Thus, in future studies, *qSPP4* and *qGW9* should be first found as the candidate genes and map cloned to elucidate their molecular mechanisms regulating the grain number or grain size. Then, eight newly detected QTLs should be further finely mapped. Finally, SSSLs carrying favorable alleles such as *qPN9*, *qGL4*, *qGW4*, and *qGW9* should be further used to breed new restorer lines by crossing with other SSSLs-Xihui18 carrying blast resistance genes such as *Pi1*, *Pi9*, etc. with the MAS method. The results of this study provide a good basis for the further functional analysis and breeding by design of these QTL.

## 4. Materials and Methods

### 4.1. Experimental Materials

**Figure 8:**
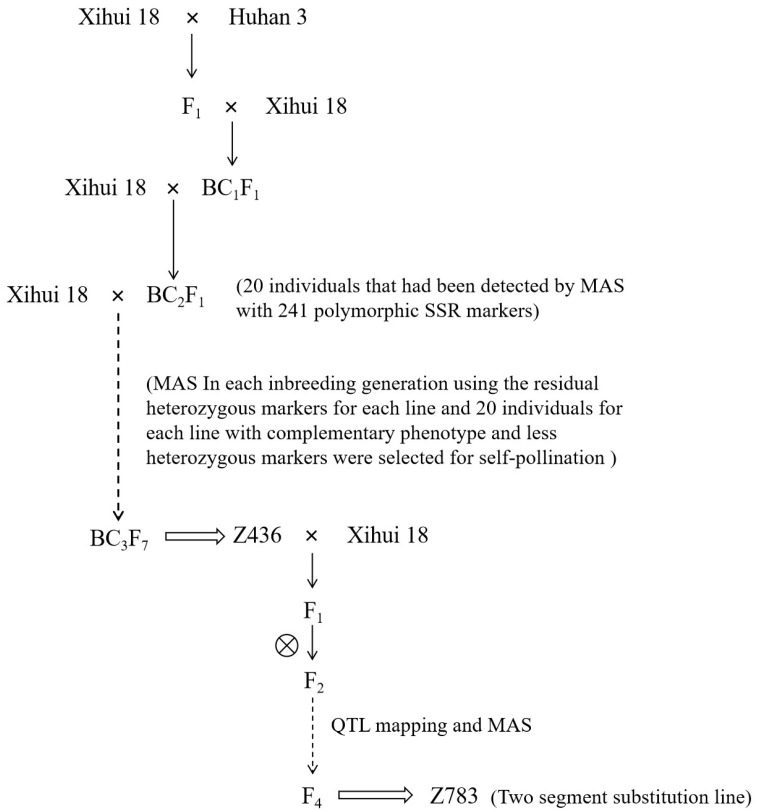
Development process of Z783 materials.

CSSL-Z783 was produced from the crossing progeny of the recipient parent Xihui18 and Z436 according to QTL mapping result and molecular marker-assisted selection (MAS) (Figure 8). Z436 was identified as carrying 8 substitution segments from the donor Huhan3 in the genetic background of Xihui18, which was developed from Xihui18 as recipient parent and Huhan3 as donor parent by advanced backcrosses and selfing in combination with SSR MAS from the BC_2_F_1_ to BC_3_F_7_ generations. The specific breeding methods description was same with development of CSSL of Z436 [38]. Chromosome substitution segments identification and the estimated lengths of the chromosome substitution segments were calculated as described by Paterson et al. [47]. The genetic map was made by the Map chart 2.32 program.

### 4.2. Material Planting Methods

Xihui18 was crossed with Z783 to obtain hybrid seeds at the experimental station (Xiema, Beibei distinct at 106.38° east longitude and 29.76° north latitude) of Southwest University, Chongqing, China, in July 2020. The hybrid seeds were planted at the Lingshui base (at 109.86° east longitude and 18.42° north latitude) in Hainan Province in September of the same year, and the F_1_ seeds were harvested. On 12 March 2021, seeds of Z783, Xihui18, and the F_2_ population of 150 plants were sown at the experimental station of Southwest University. Thirty seedlings of each parental line and all F_2_ individuals were transplanted to the field on 18 April, with 26.50 cm spacing between rows, 16.5 cm between hills, and 10 plants per row. On 10 March 2022, Three F_2_ individuals for development of SSSLs, Xihui18, and Z783 were planted in Chongqing, again with 30 individuals for the parent transplanted per line. In addition, two F_3_ populations were planted for fine-mapping of *qGW9* and *qSPP4*, and all plants were transplanted at the same experimental station in Chongqing. On 10 March 2023, 3 SSSLs for QTL identification, together with Xihui18 and Z783 were planted in Chongqing, again with 30 individuals transplanted per line. In addition, 8 secondary heterozygous F_3_ individuals for development of SSSLs together with 2 NCLs (all bands of markers same with Xihui18) were planted to use for overlapping substitution mapping of *qSPP4* and *qGW9*, each line with 100 plants. The management of the field experiments was in accordance with local standard practices.

### 4.3. Assessment of Agronomic Traits

Grains from 10 plants of Xihui18, Huhan3, Z783, SSSLs, and 150 plants of the F_2_ population were harvested at maturity. Thirteen agronomic traits were investigated, comprising plant height, panicle number per plant, panicle length, number of primary branches, number of secondary branches, spikelet number per panicle and grain number per panicle, seed setting rate, grain width, grain length, ratio of length to width, 1000-grain weight, and yield per plant. The specific methods referred to that of Zhao et al. [48]. In addition, simple statistical analysis including the mean value of each trait, standard deviation, Student’s *t*-test for comparison among ten traits betweenXihui18 and Z983, and frequency distribution of main spikelets and grain width in the F_3_ population, together with the chi-square test, was performed using statistical functions in Microsoft Exce2016.

### 4.4. QTL Mapping

For QTL mapping, 150 plants from the secondary F_2_ population were examined. DNA from each sample was extracted by using the cetyltrimethyl ammonium bromide (CTAB) technique PCR amplification, PAGE and rapid silver staining were performed using the method described by Zhao et al. [48]. Xihui18 bands were scored as “−1”, Z783 bands were scored as “1”, heterozygous bands were scored as “0”, and the absence of marker bands were scored as “.”. The marker assignments for all markers on the substitution segments of Z783, together with mean values of each trait from 150 plants in the F_2_ population, were used for QTL mapping. Mapping of QTLs was performed using the restricted maximum likelihood (REML) method implemented in the HPMIXED procedure of SAS statistical software (SAS Institute Inc., Cary, NC, USA) (http://suportsus.com/publishing (accessed on 21 June 2023), 2009, SAS/STAT: Users’ Guide, Version 9.3) with significance determined at α = 0.05.

### 4.5. Development of SSSLs

Based on the results of QTL mapping, three individuals with the target segment and few heterozygous markers were selected from the F_2_ population were used to develop SSSLs by MAS. Each individual was planted as a line with 30 plants in 2022. Then, the leaves of 20 individuals per line were collected and used to extract DNA for genotyping by MAS using both the target substitution markers and residual heterozygous markers. The SSSLs were developed based on the rule that each substitution line contained only the homozygous target substitution segment, while the bands of the other markers were the same as those of Xihui18 [39].

### 4.6. Identification and Additive Effect Analysis of QTL by Three SSSLs

In the fall of 2023, ten plants of the recipient parent Xihui18, and SSSLs (S1, S2, and S3) were harvested and measured for thirteen agronomic traits. Since there is only one chromosome segment that differs between each SSSL and Xihui18, it can be assumed that the genetic traits exhibited by the single segment substitution lines that differ from those of Xihui18 are associated with differences in this substitution segment. The genetic model of Xihui18 was *P*_0_ = *μ*_0_ + ε, and the genetic model of SSSL carrying a specific QTL was *P_i_* = *μ*_0_ + *a_i_* + *ε*, where *μ*_0_ was the phenotypic value of Xihui18 for a particular trait, *a_i_* was the additive effect of the QTL, *P_i_* and *P*_0_ denoted the phenotypic values of SSSL and Xihui18, respectively, and *ε* was the random error. For each SSSL_i_ (S1–S3), given the hypothesis (*H_0_*) that no QTL existed in the substitution segment of SSSL_i_. When *p* value < 0.05 by one-way ANOVA and LSD multiple comparisons for each SSSL_i_ in IBM SPSS Statistics 23.0, we rejected the hypothesis and argued that a QTL for a certain trait presented in the SSSL_i_ [39]. Thus, the additive effect of the phenotypic QTL can be estimated as *a_i_* = (*P_i_* − *P*_0_)/2 (half of the phenotypic difference was estimated to be caused by inheritance).

### 4.7. Fine-Mapping and Overlapping Substitution Mapping of qGW9 and qSPP4

For *qGW9*, an F_3_ population consisting of 539 plants constructed from an F_2_ recombinant individual of *qGW9* was used for genetic analysis and fine-mapping of *qGW9*. Among them, 128 recessive plants with narrow grains and one new synthesized polymorphism SSR marker together with two substitution SSR markers within the primary mapping of *qGW9* were used to analyze linkage. In addition, 5 individuals harboring different genotypes were selected for developing secondary SSSLs of *qGW9* in F_3_ population of fine-mapping. Then, the grain width of all the developed SSSL individuals together with 1 NCL population (10 plants) (all bands of markers same with Xihui18) were measured to be used for overlapping substitution mapping of *qGW9*. When grain width significantly differed between a secondary SSSL and Xihui18, a QTL for grain width was detected in the substitution segment of the SSSL. When multiple substitution segments in SSSLs with the target trait overlapped, the QTL was localized to the overlapping region [49].

For *qSPP4*, an F_3_ population consisting of 99 plants constructed from an F_2_ recombinant individual of *qSPP4* was used for genetic analysis and fine-mapping of *qSPP4*. Among them, 28 recessive plants with multiple grains and 5 new synthesized polymorphism SSR markers together with one substitution SSR marker within the primary mapping of *qSPP4* were used to analyze linkage. In addition, 3 individuals containing different genotypes were selected for the development of secondary SSSLs of *qSPP4* in an F_3_ population with fine-mapping. Then, the spikelet number of the main panicle all the developed SSSL individuals together with 1 NCL population (10 plants) (all bands of markers same with xihui18) were measured to be used for substitution mapping of *qSPP4*. The specific method of overlapping substitution mapping of *qSPP4* is the same as the above description.

## 5. Conclusions

A great deal of SSSLs in the Xihui18 genetic background are being developed from various rice donors. To date, the single-substitution segments on Chromosome 4 and Chromosome 9 have not been dissected. In this study, a CSSL Z783 was identified to harbor two substitution segments on Chr.4 and Chr.9 derived from Huhan3, with an average substitution length of 6.55 Mb. Z783 mainly displayed a higher number of panicles per plant, a wider and larger grain, and a lower number of grains per panicle compared with Xihui18. Then, nine QTLs were identified and distributed on Chromosomes 4 and 9 of Z783, and then they were dissected into three SSSLs. All the above nine QTLs could be validated by SSSLs and an additional 15 QTLs for yield-related traits were detected by S1–S3. Among them, eight QTLs including *qNSB4*, etc., might have been previously unreported compared with the reported QTLs. In addition, *qSSP4* for less grains and *qGW9* for a wide grain all displayed single gene dominant action in S2 and S3. And they were fine-mapped to smaller intervals. The research findings have laid a solid foundation for the functional analysis of *qGW9* and *qSSP4*, as well as for perfecting the library of SSSLs-Xihui18 to provide novel QTLs for breeding by design. Among these QTLs on Chr.4 and Chr.9, *qPN9* for increasing the panicle number, and *qGL4* and *qGW9* for increasing the grain size will have important roles in the improvement of rice yield.

## Figures and Tables

**Table 1:** QTL for yield-related traits identified by F_2_ population of Xihui18/Z783.

Traits.	QTL	Chr.	Linked Marker	Additive Effect	Var. (%)	*p*-Value
Panicle length (cm)	*qPL4*	4	RM3317	−0.51	12.13	0.0338
Number of secondary branches	*qNSB4*	4	RM3317	−2.84	13.07	0.0104
	*qNSB9*	9	RM2144	−2.51	10.27	0.0375
Spikelet number per panicle	*qSPP4*	4	RM3317	−10.75	8.05	0.0429
	*qSPP9*	9	RM2144	−11.78	9.67	0.0429
Grain number per panicle	*qGPP4*	4	RM3317	−11.92	10.51	0.0202
Grain width (mm)	*qGW9*	9	RM2144	0.04	11.60	0.0201
Ratio of length to width	*qRLW9*	9	RM2144	−0.04	17.96	0.0064
Yield (g)	*qYD4*	4	RM3317	−3.93	12.96	0.0096

## Data Availability

Data are contained within the article.
